# Complement Levels at Admission Reflecting Progression to Severe Acute Kidney Injury (AKI) in Coronavirus Disease 2019 (COVID-19): A Multicenter Prospective Cohort Study

**DOI:** 10.3389/fmed.2022.796109

**Published:** 2022-04-29

**Authors:** Brandon M. Henry, György Sinkovits, Ivan Szergyuk, Maria Helena Santos de Oliveira, Giuseppe Lippi, Justin L. Benoit, Emmanuel J. Favaloro, Naomi Pode-Shakked, Stefanie W. Benoit, David S. Cooper, Veronika Müller, Zsolt Iványi, János Gál, Marienn Réti, László Gopcsa, Péter Reményi, Beáta Szathmáry, Botond Lakatos, János Szlávik, Ilona Bobek, Zita Z. Prohászka, Zsolt Förhécz, Dorottya Csuka, Lisa Hurler, Erika Kajdácsi, László Cervenak, Blanka Mező, Petra Kiszel, Tamás Masszi, István Vályi-Nagy, Zoltán Prohászka

**Affiliations:** ^1^Division of Nephrology and Hypertension, Cincinnati Children's Hospital Medical Center, Cincinnati, OH, United States; ^2^The Heart Institute, Cincinnati Children's Hospital Medical Center, Cincinnati, OH, United States; ^3^Department of Pediatrics, University of Cincinnati College of Medicine, Cincinnati, OH, United States; ^4^Disease Intervention and Prevention and Population Health Programs, Texas Biomedical Research Institute, San Antonio, TX, United States; ^5^Department of Internal Medicine and Haematology, Semmelweis University, Budapest, Hungary; ^6^Faculty of Medicine, Jagiellonian University Medical College, Krakow, Poland; ^7^Department of Statistics, Maringá State University, Maringá, Brazil; ^8^Section of Clinical Biochemistry, University of Verona, Verona, Italy; ^9^Department of Emergency Medicine, University of Cincinnati College of Medicine, Cincinnati, OH, United States; ^10^Haematology, Sydney Centres for Thrombosis and Haemostasis, Westmead Hospital, Institute of Clinical Pathology and Medical Research (ICPMR), NSW Health Pathology, Westmead, NSW, Australia; ^11^Edmond and Lily Safra Children's Hospital, Sheba Medical Center, Tel-Hashomer, Israel; Sackler Faculty of Medicine, Tel-Aviv University, Tel Aviv, Israel; ^12^Department of Pulmonology, Semmelweis University, Budapest, Hungary; ^13^Department of Anaesthesiology and Intensive Therapy, Semmelweis University, Budapest, Hungary; ^14^Department of Haematology and Stem Cell Transplantation, Central Hospital of Southern Pest National Institute of Haematology and Infectious Diseases, Budapest, Hungary; ^15^Department of Infectology, Central Hospital of Southern Pest National Institute of Haematology and Infectious Diseases, Budapest, Hungary; ^16^Department of Anaesthesiology and Intensive Therapy, Central Hospital of Southern Pest National Institute of Haematology and Infectious Diseases, Budapest, Hungary; ^17^Research Group for Immunology and Haematology, Semmelweis University - Eötvös Loránd Research Network (Office for Supported Research Groups), Budapest, Hungary

**Keywords:** complement system, coronavirus disease 2019, SARS-CoV-2, acute kidney injury, renal replacement therapy (RRT)

## Abstract

**Background:**

Dysregulation of complement system is thought to be a major player in development of multi-organ damage and adverse outcomes in patients with coronavirus disease 2019 (COVID-19). This study aimed to examine associations between complement system activity and development of severe acute kidney injury (AKI) among hospitalized COVID-19 patients.

**Materials and Methods:**

In this multicenter, international study, complement as well as inflammatory and thrombotic parameters were analyzed in COVID-19 patients requiring hospitalization at one US and two Hungarian centers. The primary endpoint was development of severe AKI defined by KDIGO stage 2+3 criteria, while the secondary endpoint was need for renal replacement therapy (RRT). Complement markers with significant associations with endpoints were then correlated with a panel of inflammatory and thrombotic biomarkers and assessed for independent association with outcome measures using logistic regression.

**Results:**

A total of 131 hospitalized COVID-19 patients (median age 66 [IQR, 54–75] years; 54.2% males) were enrolled, 33 from the US, and 98 from Hungary. There was a greater prevalence of complement over-activation and consumption in those who developed severe AKI and need for RRT during hospitalization. C3a/C3 ratio was increased in groups developing severe AKI (3.29 vs. 1.71; *p* < 0.001) and requiring RRT (3.42 vs. 1.79; *p* < 0.001) in each cohort. Decrease in alternative and classical pathway activity, and consumption of C4 below reference range, as well as elevation of complement activation marker C3a above the normal was more common in patients progressing to severe AKI. In the Hungarian cohort, each standard deviation increase in C3a (*SD* = 210.1) was independently associated with 89.7% increased odds of developing severe AKI (95% CI, 7.6–234.5%). Complement was extensively correlated with an array of inflammatory biomarkers and a prothrombotic state.

**Conclusion:**

Consumption and dysregulation of complement system is associated with development of severe AKI in COVID-19 patients and could represent a promising therapeutic target for reducing thrombotic microangiopathy in SARS-CoV-2 infection.

## Introduction

Immune dysregulation has been described as a pivotal factor in development of multi-organ injury and adverse outcomes in patients with coronavirus disease 2019 (COVID-19) (1, 2), and over activation of the complement system appears to be a significant element of this maladaptive host immune response (3–7). Several reports have found elevated levels of circulating C5a, a potent anaphylatoxin, in patients with severe COVID-19 (3, 5), while deposits of an array of complement constituents have been observed in lung biopsies of patients with evidence of microangiopathic organ injury as well as in those who died from the infection (4, 5). Holter et al. demonstrated that C4d and sC5b-9 independently predicted progression to respiratory failure over the course of hospitalization for COVID-19 (7).

Leading up to the present study, our groups have conducted two separate observational studies assessing the role of complement in COVID-19. In the first, we measured a panel of complement biomarkers in 52 COVID-19 patients in the US and found elevated complement dysregulation markers C3a, C3a/C3, and sC5b-9/C3 ratios (i.e., reflecting complement over-activation and consumption) in those with severe COVID-19 at admission. These findings suggested that complement levels reflect, and may in part mediate, acute disease status (6). Whilst in another study by our group from Hungary, we similarly measured complement in a cohort of 128 COVID-19 patients, and observed that higher C3a and C3a/C3 values were associated with increased risk of in-hospital mortality in COVID-19 patients (8).

The complement cascade has an important physiological role within the innate immune system in terms of clearing invading pathogens via its many constituents, including the opsonins (C3b and C4b), the anaphylatoxins (C3a and C5a), and finally the membrane attack complex (C5b-9, MAC) (9). However, uncontrolled complement hyper-activation has been reported to contribute to vascular thrombosis and organ injury in the setting of thrombotic microangiopathies (TMAs) such as atypical hemolytic uremic syndrome (10), as well as in systemic lupus nephritis (11), antineutrophil cytoplasmic antibodies (ANCA)-associated vasculitis (12), and recently in COVID-19 (4, 13). Due to the presence of a fenestrated endothelium, the kidneys are particularly vulnerable to complement mediated attack (14). A recent observational study has reported findings of secondary TMA-mediated acute kidney injury (AKI) among COVID-19 patients, particularly when accompanied by a low ADAMTS13 (a disintegrin and metalloproteinase with a thrombospondin type 1 motif, member 13) to vWF:Ag (von Willebrand factor antigen) ratio (15). Moreover, a bidirectional relationship between vWF and complement components Factor H and C3b has been noted, such that the coagulation and complement systems can act in synergy for accelerating thrombosis (16, 17). In accordance with all these findings, it is plausible that complement is involved in the pathogenesis of microvascular thrombosis within the kidneys of COVID-19 patients leading to severe AKI.

Therefore, this prospective, international, multi-center cohort study aimed to examine the associations between complement and development of severe AKI among COVID-19 patients hospitalized in three different centers. We decided to combine the cohorts of our two individual studies, both to increase statistical power of results and account for the fact that incidence of severe COVID-19 associated AKI is highly variable between patients, hospitals, and geographical regions. In a pooled analysis, the incidence of severe AKI (defined as Kidney Disease Improving Global Outcomes [KDIGO] Stages 2+3) in COVID-19 patients averaged to ~16% (18), ranging from as little as 2.6% in China (19), up to 65% in France (20). This heterogeneity may be attributed to differences in underlying patient characteristics, varying disease course, and even under-recognition of AKI in centers with less frequent assessment of kidney function (18).

## Materials and Methods

### Study Design and Cohort

Adult patients with severe acute respiratory syndrome coronavirus 2 (SARS-CoV-2) infection confirmed by reverse transcriptase polymerase chain reaction (RT-PCR) via nasopharyngeal swab, hospitalized at the University of Cincinnati Medical Center (UCMC) in the US and two tertiary hospitals in Budapest, Hungary between April 2020 and July 2020 were consecutively enrolled in this prospective cohort study. Exclusion criterion was the presence of stage 5 chronic kidney disease (CKD) on admission.

This study was approved by the Institutional Review Board (IRB) of the University of Cincinnati (IRB ID 2020-0278) and the Hungarian Ethical Review Agency (ETT-TUKEB; No. IV/4403-2/2020/EKU) and received a waiver of informed consent in the US due to no greater than minimal risk to participants, while in the Hungarian cohort, written informed consent was obtained for each patient. This study was conducted in accordance with the Declaration of Helsinki, under the terms of relevant local and national legislation.

### Endpoints

The primary endpoint was development of severe AKI during hospitalization as defined by KDIGO Stage 2+3 serum creatinine (SCr) criteria (21). The secondary endpoint was need for renal replacement therapy (RRT). Patients were stratified for the purpose of analysis based on center of enrollment: patients from UCMC were stratified into the US cohort, while those from the two hospitals in Budapest were stratified into the Hungarian cohort.

### Measurements

Samples were collected in both cohorts at time of hospital admission. US cohort blood samples were collected at the time of a clinically indicated laboratory draw in the emergency department. Serum and EDTA-anticoagulated plasma samples were processed according to manufacturers' recommendations for complement testing and analyzed by the Clinical Nephrology Lab at Cincinnati Children's Hospital Medical Center, a national referral center for complement testing. Serum levels of 50% hemolytic complement activity (CH50), representing total complement activity (MicroVue, Quidel Corporation, San Diego, CA), and alternative pathway activity (Wieslab, SVAR, Malmö, Sweden) were assessed using enzyme-linked immunosorbent assays (ELISAs). The ELISA kits were also used to measure complement components, including C3a and sC5b-9 (MicroVue, Quidel, San Diego, CA, USA) in EDTA plasma samples. The antigen concentration of C3 and C4 was measured by immunonephelometry on a Behring Nephelometer II (BNII; Siemens Medical Solutions USA, Malvern, PA). Radioimmunoassays were performed using both institutionally developed (Factor I: Cincinnati Children's Hospital Medical Center) and commercially available antisera (Factor B, Factor H, C1q: Complement Technology, Inc., Tyler, TX). Reference values and cut-offs were defined according to manufacturers' guidelines.

Hungarian cohort blood samples (native- and EDTA-anticoagulated blood) were taken upon hospital admission, processed immediately as per standard complement testing protocols, and subsequently analyzed in their respective hospital laboratories. Identical methods were used to quantify total activity of alternative pathway, and levels of sC5b-9 and C3a, as in the US center. In Hungarian centers, total classical pathway activity was measured by hemolytic titration test based on Mayer's method (22). Antigen concentration of C3 and C4 was measured by immunoturbidimetry (Beckman Coulter, Brea, CA). Radial immunodiffusion was performed for quantifying the antigen concentrations of Factor I and Factor B, using specific polyclonal antibodies (23). The levels of Factor H and C1q were determined by homemade ELISA (23, 24). Reference values and cut-offs were defined according to manufacturers' guidelines.

### Data Collection

Data on the patient demographics, baseline characteristics, routine laboratory tests, disease course, and outcomes were extracted from patients' electronic medical records by a research professional, with select records checked for accuracy by a second investigator. Data on clinical course of admitted patients were collected through discharge/death.

### Statistical Analysis

Categorical variables were described by absolute (n) and relative (%) frequencies, and differences between AKI and RRT groups were evaluated using Fisher's exact test. For contingency tables with a number of rows or columns <2, the network algorithm developed by Mehta and Patel was used to perform the test (25). Continuous data were reported as median and interquartile range (IQR), and differences between AKI and RRT groups were analyzed using Mann-Whitney's *U* test. Additionally, C3a/C3 ratio was calculated to express the extent of overactivation and consumption of the central component of all complement pathways. Since the reference ranges and measures are different between centers, complement measurements were treated categorically in the analysis, grouped as follows: below, within, or above reference range, as defined by the individual center of testing, to enable pooled analyses. Based on results of bivariate analysis, variables with *p* < 0.010 were initially included in multivariable logistic regression to identify complement variables independently associated with development of severe AKI after adjusting for age, sex, and comorbidities, with calculation of odds ratios (OR) and 95% confidence intervals (95% CI). In regression, complement parameters were standardized to have a mean of zero and a standard deviation (SD) of one, such that a one-unit increase would correspond to an increase of one SD. Variable selection was performed via step-by-step (forward and backward) stepwise algorithm. Complement parameters with a statistically significant difference between groups were assessed for correlation with other circulating biomarkers using Spearman's correlation, and independent associations were identified through multiple step-by-step stepwise linear regression. Statistical analysis was performed using R software (version 4.0.2, R Foundation for Statistical Computing, Vienna, Austria), setting *p* < 0.05 as threshold of significance.

## Results

A total number of 135 adult hospitalized patients with RT-PCR confirmed SARS-CoV-2 infection were initially enrolled, though 4 were excluded from the analysis due to having stage 5 CKD present on admission. Thus, the final sample consisted of 131 patients: 33 in the US cohort and 98 in the Hungarian cohort. A study enrolment flow diagram for both cohorts can be found in Supplementary Figure 1.

At the time of sample collection, 38 patients (29%) required intensive care indicating a severe disease state, with 30 (22.9%) requiring invasive ventilation. Specifically, in the US Cohort (*n* = 33), 6 (18.2%) patients required intensive care at sampling, 3 (9.1%) of which required invasive ventilation. In the Hungarian cohort (*n* = 98), 32 (32.7%) patients required intensive care at sampling, 27 (27.6%) of which needed invasive ventilation. The number of patients requiring intensive care was comparable between groups (*p* = 0.13). However, there was a statistically significant difference in the need for invasive ventilation between groups (*p* = 0.03).

In the US cohort, the median (IQR) time between symptom onset and sampling was 7 (2.5–10) days. Whereas, for the Hungarian cohort, the median (IQR) time from symptom onset to sampling was 9 (6–21) days. Symptom onset date could not be determined for 16 patients in the Hungarian cohort.

### Outcomes and Patient Characteristics

Baseline patient characteristics and comorbidities are presented in Table 1. Severe AKI developed in 17.6% (*n* = 23) of the combined cohort, with 12 (9.2%) needing RRT. Overall, the median age was 66 (IQR, 54–75) years, and males amounted to 54.2% (*n* = 71) of the cohort. There were no significant differences in sex distribution between those developing/not developing severe AKI, nor those needing/not needing RRT. However, patients who developed severe AKI were significantly older (70 vs. 65 years; *p* = 0.03), while no age difference was observed among those requiring/not requiring RRT (67 vs. 65 years, *p* = 0.54). Among comorbidities, hypertension (*n* = 89; 67.9%), heart disease (*n* = 50; 38.2%), and CKD (*n* = 53; 40.5%) were the most prevalent, and both hypertension (*p* = 0.05) and CKD (*p* = 0.04) were more prevalent in severe AKI patients. Body mass index was not recorded in the Hungarian Cohort but was elevated in all US patients (median 28.5 kg/m^2^; IQR, 24.9–33.7 kg/m^2^) and significantly lower among those developing severe AKI (24.5 vs. 29.5 kg/m^2^; *p* = 0.02).

**Table 1:** Baseline characteristics (A) and comorbidities (B) in hospitalized COVID-19 patients in US and Hungary, stratified according to development of severe AKI.

	**Combined cohort**				**US cohort**				**Hungarian cohort**			
	**Total (*n =* 131)**	**No Severe AKI** **(*n =* 108)**	**Severe AKI** **(*n =* 23)**	***p***	**Total (*n =* 33)**	**No Severe AKI** **(*n =* 21)**	**Severe AKI** **(*n =* 12)**	***p***	**Total (*n =* 98)**	**No Severe AKI** **(*n =* 87)**	**Severe AKI** **(*n =* 11)**	***p***
***A. Baseline characteristics***												
Age, years	66 (54–75)	65 (53–73)	70.5 (66–78)	**0.03**	65 (47–70)	64 (40–69)	66 (56–70)	0.17	67 (56–76)	65 (54–75)	78 (73–79)	**0.002**
**Gender**												
Male	71 (54.2%)	58 (53.7%)	13 (56.5%)	0.82	18 (54.5%)	11 (52.4%)	7 (58.3%)	0.9	53 (54.1%)	47 (54.0%)	6 (54.5%)	0.9
Female	60 (45.8%)	50 (46.3%)	10 (43.5%)		15 (45.5%)	10 (47.6%)	5 (41.7%)		45 (45.9%)	40 (46.0%)	5 (45.5%)	
BMI, kg/m^2^	-	-	-	-	28.5 (24.9–33.7)	29.5 (25.8–34.5)	24.5 (21.6–27.5)	**0.02**	-	-	-	-
***B. Comorbidities***												
Obesity	-	-	-	-	18 (54.5%)	16 (76.2%)	2 (16.7%)	0.09	-	-	-	-
Hypertension	89 (67.9%)	69 (63.9%)	20 (87.0%)	**0.05**	26 (78.8%)	15 (71.4%)	11 (91.7%)	0.22	63 (64.3%)	54 (62.1%)	9 (81.8%)	0.32
Heart disease	50 (38.2%)	37 (34.3%)	13 (56.5%)	0.06	17 (51.5%)	6 (28.6%)	11 (91.7%)	**0.001**	33 (33.7%)	31 (35.6%)	2 (18.2%)	0.33
Hyperlipidemia	-	-	-	-	15 (45.5%)	11 (52.4%)	4 (33.3%)	0.73	-	-	-	-
Diabetes	43 (32.8%)	34 (31.5%)	9 (39.1%)	0.47	21 (63.6%)	15 (71.4%)	6 (50.0%)	0.74	22 (22.4%)	19 (21.8%)	3 (27.3%)	0.71
COPD	30 (22.9%)	24 (22.2%)	6 (26.1%)	0.79	8 (24.2%)	4 (19.0%)	4 (33.3%)	0.08	22 (22.4%)	20 (23.0%)	2 (18.2%)	0.9
Chronic kidney disease	53 (40.5%)	39 (36.1%)	14 (60.9%)	**0.04**	6 (18.2%)	1 (4.8%)	5 (41.7%)	**0.002**	47 (47.9%)	38 (43.7%)	9 (81.8%)	**0.02**
Chronic liver disease	-	-	-	-	7 (21.2%)	3 (14.3%)	4 (33.3%)	**0.04**	-	-	-	-

*All continuous data presented as median (IQR), with P-values calculated using Mann-Whitney's U test. All categorical data presented as frequency (%), with P-values calculated using Fisher's exact test. KDIGO, Kidney Disease Improving Global Outcomes criteria used to define AKI using serum creatinine. No severe AKI-KDIGO 0+1; Severe AKI- KDIGO 2+3. Data on BMI, obesity, hyperlipidemia, and chronic liver disease was not recorded in Hungarian cohort. Statistical significance denoted with bold text*.

### Complement Profile

A summary of complement parameters in COVID-19 patients, with levels of complement defined as below, within, or above their reference range (defined by respective testing centers), in total and stratified according to cohort and severe AKI status is shown in Table 2. Continuous measurements of complement parameters are summarized in Supplementary Table 1.

**Table 2:** Frequency of COVID-19 patients with levels of complement parameters below, within, or above their respective reference range, in total and stratified according to cohort and AKI group.

**Complement parameter**		**Combined frequencies**				**US cohort**						**Hungarian cohort**					
		**Total** **(*n =* 131)**	**No severe** **AKI** **(*n* = 108)**	**Severe** **AKI** **(*n =* 23)**	***p***	**Reference range**		**Total** **(*n =* 33)**	**No severe** **AKI** **(*n =* 21)**	**Severe** **AKI** **(*n =* 12)**	***p***	**Reference range**		**Total** **(*n =* 98)**	**No severe AKI** **(*n =* 87)**	**Severe** **AKI** **(*n =* 11)**	***p***
Alternative pathway	Below	32	23 (21.3%)	9 (39.1%)	0.18	>63%	Below	5	3 (14.3%)	2 (16.7%)	0.9	70–130%	Below	27	20 (23.0%)	7 (63.6%)	**0.03**
	Normal	97	83 (76.9%)	14 (60.9%)			Normal	28	18 (85.7%)	10 (83.3%)			Normal	69	65 (74.7%)	4 (36.4%)	
	Above	2	2 (1.9%)	0 (0.0%)									Above	2	2 (2.3%)	0 (0.0%)	
Classical pathway	Below	19	12 (11.1%)	6 (26.1%)	0.06	101–300 CH50/mL	Below	7	4 (19.0%)	2 (16.7%)	0.49	48–103 CH50/mL	Below	12	8 (9.2%)	4 (36.4%)	**0.04**
	Normal	103	89 (82.4%)	14 (60.9%)			Normal	22	15 (71.4%)	7 (58.3%)			Normal	81	74 (85.1%)	7 (63.6%)	
	Above	10	7 (6.5%)	3 (13.0%)			Above	5	2 (9.5%)	3 (25.0%)			Above	5	5 (5.7%)	0 (0.0%)	
C3	Below	16	12 (11.1%)	4 (17.4%)	0.34	71–150 mg/dL	Below	2	1 (4.8%)	1 (8.3%)	0.9	90–180 mg/dL	Below	14	11 (12.6%)	3 (27.3%)	0.49
	Normal	99	84 (77.8%)	15 (65.2%)			Normal	19	12 (57.1%)	7 (58.3%)			Normal	80	72 (82.8%)	8 (72.7%)	
	Above	16	12 (11.1%)	4 (17.4%)			Above	12	8 (38.1%)	4 (33.3%)			Above	4	4 (4.6%)	0 (0.0%)	
C3a	Below	–			**0.005**	30–250 ng/mL	Below				0.3	70–270 ng/mL	Below				**0.01**
	Normal	69	63 (58.3%)	6 (26.1%)			Normal	16	12 (57.1%)	4 (33.3%)			Normal	53	51 (58.6%)	2 (18.2%)	
	Above	60	43 (39.8%)	17 (73.9%)			Above	17	9 (42.9%)	8 (66.7%)			Above	43	34 (39.1%)	9 (81.8%)	
C4	Below	8	5 (4.6%)	3 (13.0%)	0.27	15.7–47 mg/dL	Below	1	1 (4.8%)	0 (0.0%)	0.78	15–55 mg/dL	Below	7	4 (4.6%)	3 (27.3%)	**0.04**
	Normal	107	90 (83.3%)	17 (73.9%)			Normal	26	17 (81.0%)	9 (75.0%)			Normal	81	73 (83.9%)	8 (72.7%)	
	Above	16	13 (12.0%)	3 (13.0%)			Above	6	3 (14.3%)	3 (25.0%)			Above	10	10 (11.5%)	0 (0.0%)	
C1q	Below	22	18 (16.7%)	4 (17.4%)	0.12	5.1–7.5 mg/dL	Below	16	12 (57.1%)	4 (33.3%)	0.33	6–18 mg/dL	Below	6	6 (6.9%)	0 (0.0%)	0.78
	Normal	96	82 (75.9%)	14 (60.9%)			Normal	10	6 (28.6%)	4 (33.3%)			Normal	86	76 (87.4%)	10 (90.9%)	
	Above	13	8 (7.4%)	5 (21.7%)			Above	7	3 (14.3%)	4 (33.3%)			Above	6	5 (5.7%)	1 (9.1%)	
sC5b-9	Below	3	3 (2.8%)	0 (0.0%)	0.80	<244 ng/mL					0.9	110–252 ng/mL	Below	3	3 (3.4%)	0 (0.0%)	0.82
	Normal	54	43 (39.8%)	11 (47.8%)			Normal	22	14 (66.7%)	8 (66.7%)			Normal	32	29 (33.3%)	3 (27.3%)	
	Above	72	60 (55.6%)	12 (52.2%)			Above	11	7 (33.3%)	4 (33.3%)			Above	61	53 (60.9%)	8 (72.7%)	
Factor B	Below	18	13 (12.0%)	5 (21.7%)	0.10	13.3–31.5 mg/dL	Below	14	10 (47.6%)	4 (33.3%)	0.49	70–130%	Below	4	3 (3.4%)	1 (9.1%)	0.35
	Normal	74	59 (54.6%)	15 (65.2%)			Normal	19	11 (52.4%)	8 (66.7%)			Normal	55	48 (55.2%)	7 (63.6%)	
	Above	39	36 (33.3%)	3 (13.0%)			Above						Above	39	36 (41.4%)	3 (27.3%)	
Factor H	Below	7	5 (4.6%)	2 (8.7%)	0.33	37.0–68.0 mg/dL	Below	2	2 (9.5%)	0 (0.0%)	0.63	25–88 mg/dL	Below	5	3 (3.4%)	2 (18.2%)	0.14
	Normal	77	66 (61.1%)	11 (47.8%)			Normal	15	10 (47.6%)	5 (41.7%)			Normal	62	56 (64.4%)	6 (54.5%)	
	Above	47	37 (34.3%)	10 (43.5%)			Above	16	9 (42.9%)	7 (58.3%)			Above	31	28 (32.2%)	3 (27.3%)	
Factor I	Below	12	9 (8.3%)	3 (13.0%)	0.46	2.4–4.9 mg/dL	Below				**0.03**	70–130%	Below	12	9 (10.3%)	3 (27.3%)	0.20
	Normal	91	74 (68.5%)	17 (73.9%)			Normal	22	11 (52.4%)	11 (91.7%)			Normal	69	63 (72.4%)	6 (54.5%)	
	Above	28	25 (23.1%)	3 (13.0%)			Above	11	10 (47.6%)	1 (8.3%)			Above	17	15 (17.2%)	2 (18.2%)	

*All data presented as frequencies (%). P-values calculated with Fisher's exact test. KDIGO, Kidney Disease Improving Global Outcomes criteria used to define AKI using serum creatinine. No severe AKI-KDIGO 0+1; Severe AKI-KDIGO 2+3; CH50-50% hemolytic complement activity. Statistical significance denoted with bold text*.

When looking at complement levels as categorical variables in the combined cohort, a C3a level above reference range was more common in patients with severe AKI (73.9 vs. 39.8%; *p* = 0.005). Within the Hungarian cohort, severe AKI patients more frequently had below normal alternative pathway activity (63.6 vs. 23.0%; *p* = 0.03), classical pathway activity (36.4 vs. 9.2%; *p* = 0.04), as well as above normal C4 levels (27.3 vs. 4.6%; *p* = 0.04), and C3a levels (81.8 vs. 39.1%; *p* = 0.01). In the US cohort, Factor I elevation was less prevalent in severe AKI patients (8.3 vs. 47.6%; *p* = 0.03).

**Figure 1:**
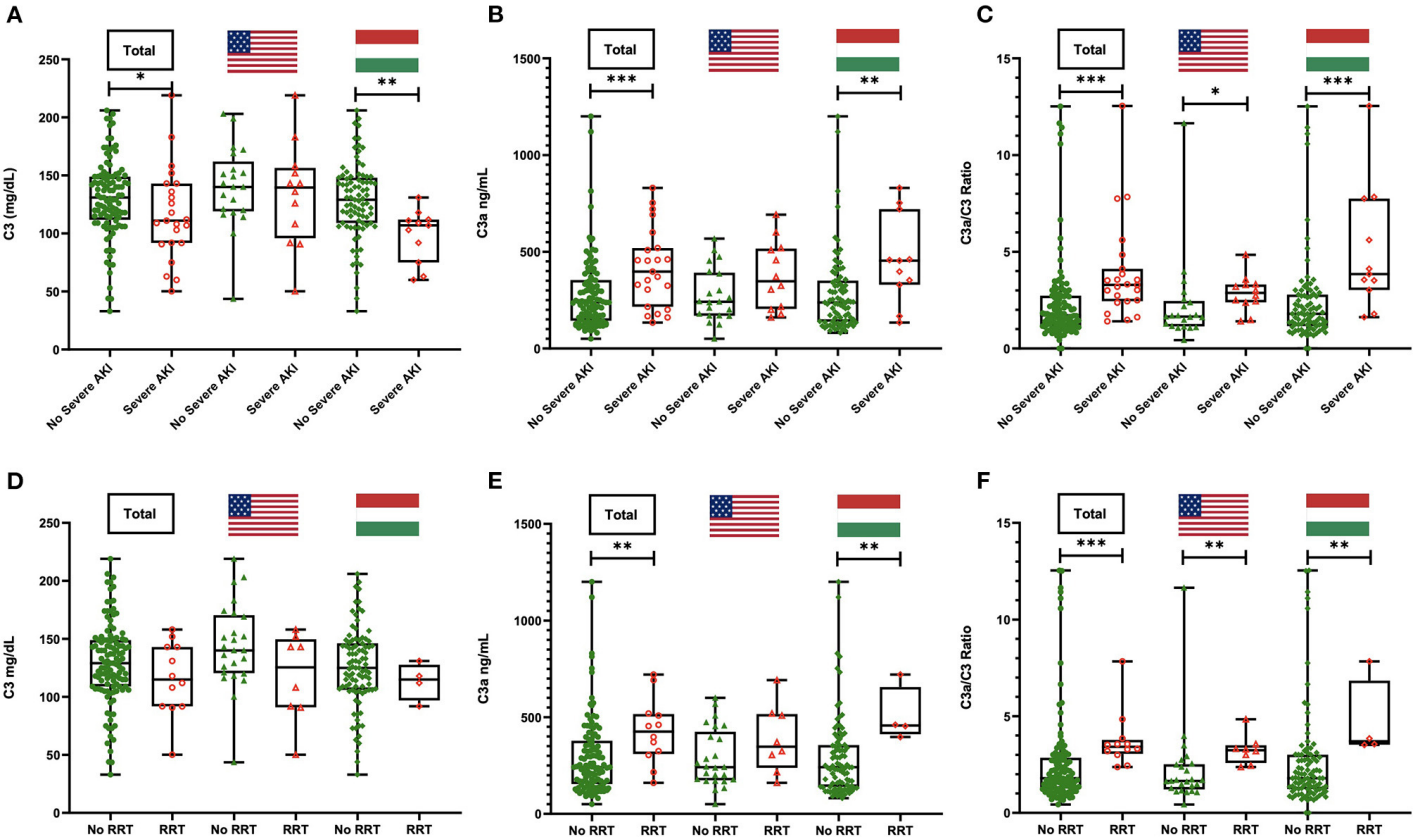
C3, C3a, and C3a/C3 ratio levels in patients with or without severe acute kidney injury (AKI) **(A–C)** and with or without need for renal replacement therapy (RRT) **(D–F)** in the total cohort and stratified by country. *P<*0.05;* **P < 0.01; ***P < 0.001.

When looking at complement levels as continuous variables in the total cohort, the C3a/C3 ratio was higher in patients with severe AKI (3.29 vs. 1.71; *p* < 0.001) (Figure 1). Among the Hungarian cohort, those who developed severe AKI had lower alternative pathway activity (*p* = 0.008), classical pathway activity (*p* = 0.001), C3 (*p* = 0.002), and C4 (*p* = 0.002), while C3a (*p* = 0.005) and C3a/C3 ratio (*p* < 0.001) were significantly elevated in these patients. Among the US cohort, only C3a/C3 ratio was significantly elevated in severe AKI patients (*p* = 0.016) (Supplementary Table 1).

Patients who needed RRT had significantly greater prevalence of C3a levels above reference range compared with those who did not (100 vs. 53.8%; *p* = 0.001). When stratified, this pattern remained significant in the Hungarian cohort only (100 vs. 42.4%; *p* = 0.04). Additionally, C3a/C3 ratio was significantly higher in patients requiring RRT in the combined cohort (3.42 vs. 1.79; *p* < 0.001), as well as in the US (3.24 vs. 1.65; *p* = 0.002) and Hungarian (3.7 vs. 1.79; *p* = 0.01) cohorts separately (Figure 1 and Table 3).

**Table 3:** Complement levels on admission in patients with COVID-19 in combined cohort, and in US and Hungary separately, stratified by need for renal replacement therapy (RRT).

**Variable**		**Combined Cohort**				**US Cohort**				**Hungarian Cohort**			
		**Total** **(*n* = 131)**	**No need** **for RRT** **(*n =* 119)**	**Need for** **RRT** **(*n =* 12)**	***p***	**Total (*n =* 33)**	**No need** **for RRT** **(*n =* 25)**	**Need for RRT (*n =* 8)**	***p***	**Total** **(*n =* 98)**	**No need** **for RRT** **(*n =* 94)**	**Need for** **RRT** **(*n =* 4)**	***p***
C3	Below	16	15 (12.6%)	1 (8.3%)	0.87	2	1 (4%)	1 (12.5%)	0.43	14	14 (14.9%)	0 (0,0%)	0.9
	Normal	99	90 (75.6%)	9 (75%)		19	14 (56%)	5 (62.5%)		80	76 (80.9%)	4 (100%)	
	Above	16	14 (11.8%)	2 (16.7%)		12	10 (40%)	2 (25%)		4	4 (4.2%)	0 (0,0%)	
C3a	Below	–			**0.001**				0.9				**0.04**
	Normal	54	54 (46.2%)	0 (0%)		1	1 (4%)	0 (0%)		53	53 (57.6%)	0 (0,0%)	
	Above	75	63 (53.8%)	12 (100%)		32	24 (96%)	8 (100%)		43	39 (42.4%)	4 (100%)	
C3a/C3		1.86 (1.27–3.02)	1.79 (1.24–2.81)	3.42 (3.15–3.63)	**<0.001**	2.18 (1.48–3.01)	1.65 (1.28–2.49)	3.24 (2.86–3.38)	**0.002**	1.84 (1.25–3.03)	1.79 (1.23–2.97)	3.7 (3.54–4.84)	**0.01**

*All continuous data presented as median (IQR), with P-values calculated using Mann-Whitney's U test. All categorical data presented as frequency (%), with P-values calculated using Fisher's exact test. RRT, renal replacement therapy. Statistical significance denoted with bold text*.

In multivariate logistic regression, adjusted for age, and comorbidities, C3a measured at admission in the Hungarian cohort predicted the development of severe AKI during hospitalization (Table 4). Each standard deviation increase in C3a (*SD* = 210.1) was independently associated with 89.7% increased odds of developing severe AKI (95% CI, 7.6–234.5%).

**Table 4:** Multivariate logistic regression for development of severe AKI over course of hospitalization in the US cohort (A) and the Hungarian cohort (B).

**Variable**	**Coefficient**	**Standard Error**	***p***	**OR (95% CI)**
***A. US cohort***				
**BMI**	−0.15967	0.07682	0.04	0.852 (0.733–0.991)
**Hypertension**	3.63947	1.23538	0.03	14.006 (1.244–157.71)
***B. Hungarian cohort***				
**Age**	0.10874	0.041809	0.009	1.115 (1.027–1.210)
**C3a (standardized)**	0.6404	0.2893	0.03	1.897 (1.076–3.345)

*OR, odds ratio; 95%CI, 95% confidence interval; BMI, body mass index*.

### Correlations and Associations With Inflammatory and Thrombotic Biomarkers

Correlation analysis was performed for complement parameters that had significant difference between groups, for the purpose of identifying interplays and associations with imbalances in other systems (Table 5). Independent associations were assessed through stepwise linear regression (Supplementary Table 2).

**Table 5:** Spearman's Correlation coefficients between complement parameters and other circulating biomarkers measured on admission.

	**Hungarian cohort**					**US cohort**	
	**Alternative Pathway**	**Classical pathway**	**C3a**	**C4**	**C3a/C3**	**Factor I**	**C3a/C3**
ED Creatinine	−0.079 (*p =* 0.44)	−0.179 (*p =* 0.08)	0.144 (*p =* 0.16)	−0.066 (*p =* 0.52)	**0.276 (*p =* 0.006)**	**−0.499 (*p =* 0.003)**	**0.696 (*p =* 0.001)**
ADAMTS13	0.491 **(*p =* 0.001)**	**0.269 (*p =* 0.007)**	−0.183 (*p =* 0.07)	**0.310 (*p =* 0.002)**	**−0.318 (*p =* 0.001)**	**0.389 (*p =* 0.03)**	**−0.398 (*p =* 0.02)**
vWF	−0.164 (*p =* 0.12)	−0.152 (*p =* 0.15)	**0.387 (*****p** **<*** **0.001)**	−0.077 (*p =* 0.47)	**0.370 (*****p** **<*** **0.001)**	0.182 (*p =* 0.31)	0.133 (*p =* 0.46)
ADAMTS13/vWF:Ag	**0.395 (*****p** **<*** **0.001)**	**0.245 (*****p** **=*** **0.02)**	**−0.353 (*****p** **<*** **0.001)**	0.259 (*p =* 0.01)	**−0.441 (*****p** **<*** **0.001)**	0.131 (*p =* 0.47)	**−0.407 (*****p** **=*** **0.02)**
CRP	0.034 (*p =* 0.74)	0.128 (*p =* 0.21)	**0.618 (*****p** **<*** **0.001)**	0.160 (*p =* 0.12)	**0.475 (*****p** **<*** **0.001)**	0.149 (*p =* 0.41)	0.284 (*p =* 0.11)
Ferritin	−0.037 (*p =* 0.73)	0.016 (*p =* 0.88)	**0.392 (*****p** **<*** **0.001)**	0.032 (*p =* 0.76)	**0.305 (*****p** **=*** **0.003)**	−0.188 (*p =* 0.30)	**0.365 (*****p** **=*** **0.04)**
IL-6	−0.130 (*p =* 0.21)	−0.028 (*p =* 0.79)	**0.340 (*****p** **<*** **0.001)**	0.067 (*p =* 0.51)	**0.298 (*****p** **=*** **0.003)**	−0.139 (*p =* 0.45)	**0.419 (*****p** **=*** **0.02)**
Fibrinogen	**0.305 (*****p** **=*** **0.01)**	**0.371 (*****p** **=*** **0.002)**	0.141 (*p =* 0.25)	**0.260 (*****p** **=*** **0.03)**	−0.026 (*p =* 0.83)	**0.537 (*****p** **=*** **0.006)**	0.021 (*p =* 0.92)
LDH	−0.119 (*p =* 0.26)	−0.161 (*p =* 0.13)	**0.289 (*****p** **=*** **0.006)**	−0.194 (*p =* 0.07)	**0.320 (*****p** **=*** **0.002)**	−0.150 (*p =* 0.41)	0.220 (*p =* 0.22)
Lymphocytes	**0.230 (*****p** **=*** **0.02)**	−0.031 (*p =* 0.76)	**−0.296 (*****p** **=*** **0.004)**	−0.144 (*p =* 0.16)	**−0.279 (*****p** **=*** **0.006)**	0.067 (*p =* 0.71)	−0.217 (*p =* 0.22)
Neutrophils	**−0.218 (*****p** **=*** **0.03)**	−0.188 (*p =* 0.07)	**0.215 (*****p** **=*** **0.04)**	−0.197 (*p =* 0.05)	**0.215 (*****p** **=*** **0.04)**	0.146 (*p =* 0.42)	0.137 (*p =* 0.44)
NLR	**−0.276 (*****p** **=*** **0.006)**	−0.110 (*p =* 0.28)	**0.345 (*****p** **<*** **0.001)**	−0.052 (*p =* 0.61)	**0.333 (*****p** **<*** **0.001)**	0.027 (*p =* 0.88)	0.218 (*p =* 0.22)
Haptoglobin	**0.204 (*****p** **=*** **0.04)**	**0.351 (*****p** **<*** **0.001)**	0.180 (*p =* 0.08)	**0.335 (*****p** **<*** **0.001)**	−0.059 (*p =* 0.56)	**0.478 (*****p** **=*** **0.005)**	−0.066 (*p =* 0.72)

*ED Creatinine, emergency department creatinine; ADAMTS13, a disintegrin and metalloproteinase with a thrombospondin type 1 motif, member 13; vWF, Ag, von Willebrand factor antigen; CRP, C reactive protein; IL-6, interleukin-6; LDH, lactate dehydrogenase; NLR, neutrophil to lymphocyte ratio; CH50, 50% hemolytic complement activity. Statistical significance denoted with bold text*.

In the US cohort, Factor I correlated positively with ADAMTS13 (*p* = 0.03), fibrinogen (*p* = 0.006), and haptoglobin (*p* = 0.005); and negatively with admission creatinine (*p* = 0.003). C3a/C3 correlated positively with admission creatinine (*p* < 0.001), ferritin (*p* = 0.04), and IL-6 (*p* = 0.02); and inversely with ADAMTS13 (*p* = 0.02) and ADAMTS13/vWF:Ag ratio (*p* = 0.02).

Factor I maintained independent positive association with haptoglobin (*p* = 0.048) and negative association with admission creatinine (*p* = 0.026) in linear regression. C3a/C3 ratio remained independently and positively associated with admission creatinine (*p* = 0.035) and IL-6 (*p* < 0.001).

In the Hungarian cohort, alternative pathway activity and classical pathway activity both correlated positively with ADAMTS13 (*p* < 0.001; *p* = 0.007), ADAMTS13/vWF:Ag ratio (*p* < 0.001; *p* = 0.02), fibrinogen (*p* = 0.01; *p* = 0.002), and haptoglobin (*p* = 0.044; *p* < 0.001). Alternative pathway activity additionally correlated positively with lymphocyte count (*p* = 0.02) and inversely with neutrophil count (*p* = 0.03) and neutrophil-to-lymphocyte ratio (NLR) [*p* = 0.006]. C3a and C3a/C3 both correlated positively with vWF:Ag (*p* < 0.001; *p* < 0.001), C reactive protein (CRP) [*p* < 0.001; *p* < 0.001], ferritin (*p* < 0.001; *p* = 0.003), interleukin (IL)-6 (*p* < 0.001; *p* = 0.003), lactate dehydrogenase (LDH) [*p* = 0.006; *p* = 0.002], neutrophil count (*p* = 0.04; *p* = 0.04), and NLR (*p* < 0.001; *p* < 0.001); and negatively with ADAMTS13/vWF:Ag ratio (*p* < 0.001; *p* < 0.001), and lymphocyte count (*p* = 0.004; *p* = 0.006). C3a/C3 ratio also correlated with admission creatinine (*p* = 0.006), and ADAMTS13 (*p* = 0.001).

Alternative pathway activity remained independently and positively associated with ADAMTS13/vWF:Ag ratio and haptoglobin (*p* = 0.011 and *p* = 0.009, respectively), and negatively with NLR (*p* = 0.007). Classical pathway activity maintained independent association with increase in fibrinogen (*p* = 0.019) and haptoglobin (*p* = 0.003), as well as decrease in neutrophil count (*p* < 0.001). C3a remained independently and positively associated with CRP (*p* < 0.001) and ferritin (*p* = 0.016), and negatively with fibrinogen (*p* = 0.034). C4 maintained positive independent association with fibrinogen (*p* = 0.003), and negative with LDH (*p* = 0.009) and neutrophil count (*p* = 0.007). Lastly, the C3a/C3 ratio remained independently associated with increase in CRP (*p* < 0.001), as well as decrease in LDH (*p* < 0.001) and neutrophil count (*p* < 0.001).

## Discussion

Overall, these findings show a greater prevalence of complement over-activation and consumption on admission in COVID-19 patients who developed severe AKI and required RRT during hospitalization. Specifically, elevations of complement activation marker and potent anaphylatoxin C3a were more frequently found in this patient population, such that each standard deviation increase in C3a was independently associated with 89.7% increased odds of developing severe AKI in logistic regression. Furthermore, C3a/C3 ratio, reflecting the magnitude of complement over-activation and consumption, was consistently increased in those developing severe AKI and requiring RRT in each cohort.

C3a was the only complement parameter independently predicting progression to severe AKI. We suspect C3a may contribute to TMA-mediated renal injury (frequently seen in COVID-19-related AKI) (26), by inducing inflammation, activating the endothelium (27), and triggering platelet aggregation by binding of the C3a receptor (C3aR) on platelets (4, 28). Consistent with these findings, we found that decreased alternative and classical pathway activity, and consumption of C4, a lectin and classical pathway component, were more common in COVID-19 patients who progressed to severe AKI. Importantly, in a recent study combining transcriptomic, proteomic, and mechanistic studies, Georg and colleagues identified that C3a induces highly cytotoxic CD16+ T cells in patients with severe COVID-19 (29). These cells cause T-cell receptor independent degranulation, cytotoxicity, and microvascular injury particularly unique to COVID-19 (29). This finding provides further evidence linking elevations in C3a to adverse outcomes in COVID-19 patients.

AKI in COVID-19 patients is associated with significantly prolonged ICU stay and increased mortality, particularly in those with severe illness (30, 31). Complement levels on admission may hence be useful for risk-stratification of COVID-19 patients. Furthermore, interventions directed at suppressing a dysregulated complement cascade, including the administration of eculizumab (anti-C5 monoclonal antibody) (32), BDB-0001 (anti-C5a monoclonal antibody) (5), and AMY-101 (a compstatin-based C3 inhibitor) (33), have shown promising potential at attenuating the complement-mediated hyper-inflammatory response and severe outcomes in COVID-19 patients in several small case series, and should be investigated in the context of reducing TMA-mediated sequalae in the kidneys. More recent studies on anti-complement therapy have largely focused on eculizumab, underpinning its safety, and efficacy at improving respiratory function and survival in COVID-19 patients (34–37). Nonetheless, it is important to note that early complement inhibitors (i.e., AMY-101) appear to offer broader therapeutic control compared to complement inhibitors targeting later stages of the complement cascade (i.e., eculizumab) (38). However, large prospective trials involving anti-complement therapy are still underway (clinicaltrials.gov, NCT04288713, NCT04395456, NCT04382755). Several recent studies identified variants of complement-related genes predisposing to more severe outcomes in COVID-19, suggesting that anti-complement therapy can be targeted at individuals with a more susceptible genotype (48–51).

Alternative and classical complement pathway activity, and particularly the complement marker C4, was found to positively correlate with ADAMTS13 and ADAMTS13/vWF:Ag ratio. Although, after multivariate regression, only alternative pathway activity maintained independent association with ADAMTS13/vWF:Ag ratio. When the components of alternative and classical pathways are consumed, as in patients developing severe AKI, this would reflect a state characteristic of TMA (15, 39). Conversely, complement activation product C3a and the C3a/C3 ratio, which were elevated in these patients, displayed a parallel inverse, although not independent, correlation with these pathognomonic TMA markers. These findings not only corroborate emerging evidence of secondary TMA-mediated AKI in COVID-19 patients (6, 15), but also suggest a parallel role of complement in the process.

Similarly, in a study by Holter et al. complement dysregulation on admission was associated with organ dysfunction, albeit pulmonary, later on in the course of illness (7). Gao et al. observed complement deposits in pulmonary tissue of patients who succumbed to COVID-19, comprising mainly C3, C4a, C5b-9, mannose binding lectin (MBL), and mannose-binding protein-associated serine protease 2 (MASP-2) (5). Furthermore, Magro et al. demonstrated evidence of C4d, MASP-2, and terminal complement (C5d-9) deposits in lung and skin tissue biopsies in patients with confirmed microvascular thrombosis (4). The researchers concluded that complement may augment thrombotic microvascular injury through endothelial damage and resultant activation of coagulation (4). In keeping with this, it is conceivable that deposition of complement activation products in the kidneys may also mediate renal injury in COVID-19 patients. It should be noted, that complement may also induce formation of neutrophil extracellular traps (NETs) (40), extracellular chromatin-based fibers that have been implicated in various thrombotic pathologies (41–43). NETs in turn provide a scaffolding on which further complement may be activated (44). This process has been demonstrated *in vitro*, wherein incubation of NETs with serum led to consumption of complement components and generation of complement activation products (45). However, NETosis was not measured in our cohorts, and its role in complement mediated renal injury in COVID-19 should be further investigated.

Lastly, C3a and C3a/C3 both positively correlated with an array of inflammatory biomarkers, including CRP, ferritin, and IL-6. The importance of this interplay is strengthened by the efficacy of anti-complement therapy to dampen a dysregulated inflammatory response as reported in several recent studies on COVID-19 (33, 38, 46). Furthermore, negative correlation with the lymphocyte count, and positive correlation with the neutrophil count as well as with NLR, points to a state of lymphopenia and neutrophilia in patients developing severe AKI. In a recent meta-analysis by Henry and colleagues, this pattern at admission has been associated with increased odds of severe disease and mortality in patients with SARS-CoV-2 infection (47).

There are a number of sources of limitations in this pooled analysis. First, with only 33 hospitalized patients, the US cohort was considerably smaller than the Hungarian cohort. However, this is largely explained by the absence of a significant first wave where our US center is located. Due to the emergent nature of the pandemic, we did not perform *a priori* sample size calculations. The paucity of significant findings in the US cohort could be type 2 error on the basis of low statistical power. Conversely, cohorts of different geographic settings may have been impacted by confounders such as distinct inflammatory patterns, underlying patient demographics, such as slightly more advanced age in the Hungarian cohort (especially among those with severe AKI) and the higher prevalence of cardiovascular comorbidities in the US cohort, as well as differences in dominant circulating viral strains (52). Similarly, due to sample limitations, we did not explore the impact of racial or ethnic background on the obtained results. Furthermore, despite the same classification, there was a difference in the incidence of underlying CKD and development of severe AKI during illness between countries in our study, with less CKD but more frequent severe AKI in the US cohort, which may be partially attributed to small cohort sizes, as well as the aforementioned confounders. Further research should employ a larger sample to reduce influence of inter-individual differences within and between cohorts and increase generalizability of results beyond the study. The laboratory methods for measurement of certain complement parameters were unique to each testing center, and each center used their own cut-off points that have been pre-established on the basis of their population. To account for this, we resorted to treating complement measurements as categorical variables, relative to their respective reference range, except in the case of the C3a/C3 ratio for which a reference range is unavailable. Finally, we only measured complement levels at time of hospital admission, which provides no indication of changes in complement profile over the course of illness or the impact of variable disease course and sample collection timing on complement parameters.

In conclusion, we found evidence of an association between complement consumption and dysregulation on admission, and progression to severe AKI and need for RRT in COVID-19 patients in our multicenter observational study. We hence suggest that C3a/C3 ratio and C3a levels upon admission could be considered potential early predictors of the risk to develop severe AKI and a potential therapeutic target in this patient population. Further research would be needed to elucidate the mechanisms and role of complement derangement in COVID-19.

## Data Availability Statement

The raw data supporting the conclusions of this article will be made available by the authors, without undue reservation.

## Ethics Statement

The studies involving human participants were reviewed and approved by Institutional Review Board (IRB) of the University of Cincinnati (IRB ID 2020-0278) and the Hungarian Ethical Review Agency (ETT-TUKEB; No. IV/4403-2/2020/EKU). Written informed consent for participation was not required for this study in accordance with the national legislation and the institutional requirements.

## Author Contributions

BH, GS, DCs, EF, GL, VM, ZI, JG, MR, LG, PR, BS, BL, JS, IB, ZiP, TM, IV-N, and ZoP: research idea and study design. BH, JB, SB, NP-S, BM, ZF, DCs, LH, EK, LC, and PK: sample collection and running experiments. JB, MO, DCs, GS, MR, VM, ZI, JG, LG, PR, BS, BL, JS, IB, ZiP, TM, and ZoP: data acquisition. BH, IS, MO, GS, ZiP, and ZoP: data analysis. BH, GS, IS, GL, JB, DCs, EF, NP-S, SB, DCo, VM, ZI, JG, MR, LG, PR, BS, BL, JS, IB, ZiP, ZF, LH, EK, LC, BM, PK, TM, and ZoP: data interpretation. IS: preparation of first draft. All authors contributed important intellectual content during manuscript drafting or revision and agrees to be personally accountable for the individual's own contributions and to ensure that questions pertaining to the accuracy or integrity of any portion of the work are appropriately investigated and resolved.

## Funding

This study was funded by the University of Cincinnati College of Medicine Special Coronavirus (COVID-19) Research Pilot Grant Program, as well as the Higher Education Institutional Excellence Program of the Ministry of Human Capacities in Hungary, within the framework of the molecular biology thematic program of the Semmelweis University, and by the National Office for Innovation and Research (KH130355 to ZoP, and 2020-1.1.6-JOVO-2021-00013 to ZoP), and by the MSCA-ITN (Horizon 2020) “CORVOS” (Grant 860044 to ZoP and LH). DCs was supported by a grant of the Premium Postdoctoral Fellowship Program of the Hungarian Academy of Sciences (PPD2018-016/2018).

## Conflict of Interest

The authors declare that the research was conducted in the absence of any commercial or financial relationships that could be construed as a potential conflict of interest.

## Publisher's Note

All claims expressed in this article are solely those of the authors and do not necessarily represent those of their affiliated organizations, or those of the publisher, the editors and the reviewers. Any product that may be evaluated in this article, or claim that may be made by its manufacturer, is not guaranteed or endorsed by the publisher.
