# Supplementary material for: Complement Levels at Admission Reflecting Progression to Severe Acute Kidney Injury (AKI) in Coronavirus Disease 2019 (COVID-19): A Multicenter Prospective Cohort Study
Source: Front Med (Lausanne). 2022 Apr 29;9:796109. doi: 10.3389/fmed.2022.796109 (PMC9100416; doi:10.3389/fmed.2022.796109)
Supplement: Supplementary file 1 [file Table_1.DOCX]

# **Table S1.** Complement levels on admission in patients with COVID-19 in US and Hungary, stratified by development of severe AKI.

| **Lab variable** | **Reference range** | **US Cohort (n=33)** | | |  | **Reference range** | **Hungarian Cohort (n=98)** | | |
| --- | --- | --- | --- | --- | --- | --- | --- | --- | --- |
|  |  | **No severe AKI (n=21)** | **Severe AKI (n=12)** | **P-value** |  |  | **No severe AKI (n=87)** | **Severe AKI (n=11)** | **P-value** |
| **Alternative pathway** | >63% | 89.5 (75.6 – 101.8) | 98.9 (80.6 – 104.4) | 0.58 |  | 70-130% | 93 (75.5-102) | 68 (58.5-83.5) | **0.008** |
| **Classical pathway** | 101 – 300 CH50/mL | 202.0 (140.0 – 252.6) | 180.7 (137.1 – 302.8) | 0.86 |  | 48-103 CH50/mL | 74 (66-88.5) | 53 (43-64) | **0.001** |
| **C3** | 71 – 150 mg/dL | 140.0 (119.0 – 162.0) | 139.5 (104.0 – 153.5) | 0.58 |  | 90-180  mg/dL | 129 (109-147) | 107 (83-111) | **0.002** |
| **C3a** | 30 - 250 ng/mL | 242.0 (169.5 – 392.0) | 347.9 (211.9 – 511.6) | 0.098 |  | 70-270 ng/mL | 238 (140-350) | 454 (341.5-591) | **0.005** |
| **C4** | 15.7 – 47 mg/dL | 29.5 (23.3 – 40.0) | 37.1 (30.0 – 45.4) | 0.15 |  | 15-55 mg/dL | 37 (26-49) | 26 (17-27) | **0.002** |
| **C1q** | 5.1-7.5 mg/dL | 4.3 (3.2 – 6.5) | 5.7 (2.4 – 7.8) | 0.30 |  | 6.0-18.0 mg/dL | 10.6 (8.6-13.5) | 11.6 (9.6-15.0) | 0.31 |
| **sC5b-9** | <244 ng/mL | 214.9 (136.2 – 318.5) | 201.4 (167.4 – 258.6) | 0.75 |  | 110-252 ng/mL | 287 (203-425) | 362 (252-466) | 0.28 |
| **Factor B** | 13.3-31.5 mg/dL | 14.7 (9.4 – 18.1) | 17.2 (11.1 – 19.6) | 0.22 |  | 70-130% | 120 (97.5-148) | 118 (81-128) | 0.16 |
| **Factor H** | 37.0 – 68.0 mg/dL | 58.7 (48.4 – 78.4) | 71.8 (49.4 – 87.0) | 0.36 |  | 25.0-88.0 mg/dL | 74.0 (49.9-102.7) | 36.8 (31.9-89.3) | 0.12 |
| **Factor I** | 2.4 – 4.9 mg/dL | 4.7 (3.9 – 5.5) | 3.9 (3.2 – 4.8) | 0.094 |  | 70-130% | 103 (84-119) | 86 (68-117) | 0.27 |
| **C3a/C3** | - | 1.65 (1.15-2.38) | 2.87 (2.42-3.30) | **0.016** |  | - | 1.79 (1.19-2.76) | 3.85 (3.27-6.68) | **<0.001** |
| All data presented as median (IQR). P-values calculated with Mann-Whitney U test. KDIGO - Kidney Disease Improving Global Outcomes criteria used to define AKI using serum creatinine. No severe AKI – KDIGO 0+1; Severe AKI – KDIGO 2+3; CH50 – 50% hemolytic complement activity. Statistical significance denoted with bold text. | | | | | | | | | |
