# Supplementary material for: Complement Levels at Admission Reflecting Progression to Severe Acute Kidney Injury (AKI) in Coronavirus Disease 2019 (COVID-19): A Multicenter Prospective Cohort Study
Source: Front Med (Lausanne). 2022 Apr 29;9:796109. doi: 10.3389/fmed.2022.796109 (PMC9100416; doi:10.3389/fmed.2022.796109)
Supplement: Supplementary file 2 [file Table_2.DOCX]

# **Table S2.** Linear regression results for complement parameters.

| **Complement parameter** | **Variable** | **Coefficient (95% CI)** | **p-value** |
| --- | --- | --- | --- |
| *A. US cohort* | | | |
| **Factor I** | ED Creatinine (mg/dL) | -0.158 (-0.297, -0.020) | 0.026 |
|  | Haptoglobin (g/L) | 0.003 (0.000, 0.005) | 0.048 |
| **C3a/C3** | ED Creatinine (mg/dL) | 0.141 (0.011, 0.271) | 0.035 |
|  | IL-6 (pg/mL) | 0.033 (0.025, 0.040) | <0.001 |
|  | | | |
| *B. Hungarian cohort* | | | |
| **Alternative Pathway** | ADAMTS13/vWF:Ag | 26.200 (6.110, 46.290) | 0.011 |
|  | NLR | -1.246 (-2.147, -0.345) | 0.007 |
|  | Haptoglobin (g/L) | 5.325 (1.346, 9.304) | 0.009 |
| **Classical Pathway** | Fibrinogen (g/L) | 3.574 (0.601, 6.546) | 0.019 |
|  | Neutrophil Count | -2.093 (-2.967, -1.219) | <0.001 |
|  | Haptoglobin (g/L) | 5.289 (1.823, 8.756) | 0.003 |
| **C3a** | Fibrinogen (g/L) | -39.278 (-75.413, -3.143) | 0.034 |
|  | CRP (mg/L) | 1.301 (0.656, 1.945) | <0.001 |
|  | Ferritin (ng/mL) | 0.025 (0.005, 0.046) | 0.016 |
| **C4** | Fibrinogen (g/L) | 0.036 (0.013, 0.060) | 0.003 |
|  | LDH (U/mL) | -0.0002 (-0.0003, -0.00004) | 0.009 |
|  | Neutrophil Count | -0.012 (-0.020, -0.003) | 0.007 |
| **C3a/C3** | CRP (mg/L) | 0.001 (0.001, 0.002) | <0.001 |
|  | LDH (U/mL) | -0.0002 (-0.0003, -0.0001) | <0.001 |
|  | Neutrophil Count | -0.018 (-0.026, -0.011) | <0.001 |
| ED Creatinine – emergency department creatinine, IL-6 – interleukin-6, ADAMTS13 - a disintegrin and metalloproteinase with a thrombospondin type 1 motif, member 13, vWF:Ag - von Willebrand factor antigen, NLR – neutrophil to lymphocyte ratio, CRP – C reactive protein, LDH – lactate dehydrogenase, CI – confidence interval. | | | |
