# Supplementary material for: Complement Levels at Admission Reflecting Progression to Severe Acute Kidney Injury (AKI) in Coronavirus Disease 2019 (COVID-19): A Multicenter Prospective Cohort Study
Source: Front Med (Lausanne). 2022 Apr 29;9:796109. doi: 10.3389/fmed.2022.796109 (PMC9100416; doi:10.3389/fmed.2022.796109)
Supplement: Supplementary file 3 [file Image_1.PDF]

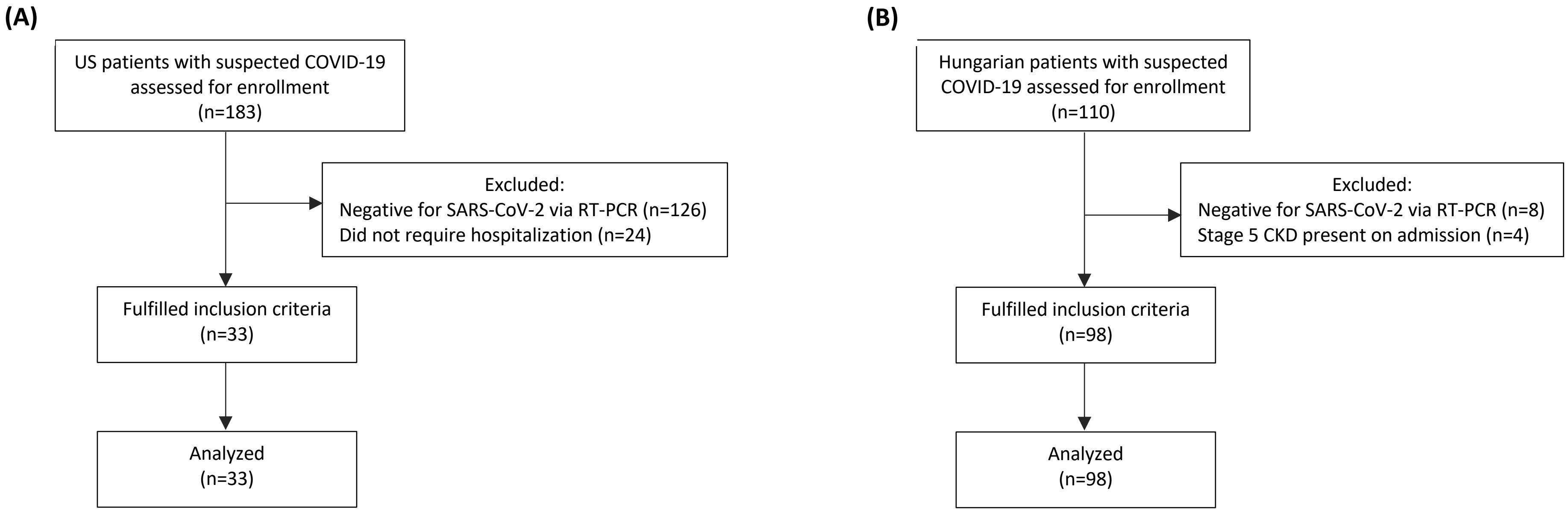

Figure S1. Study enrollment flow diagram. US cohort **(A)**, Hungarian cohort **(B)**.

Hungarian cohort

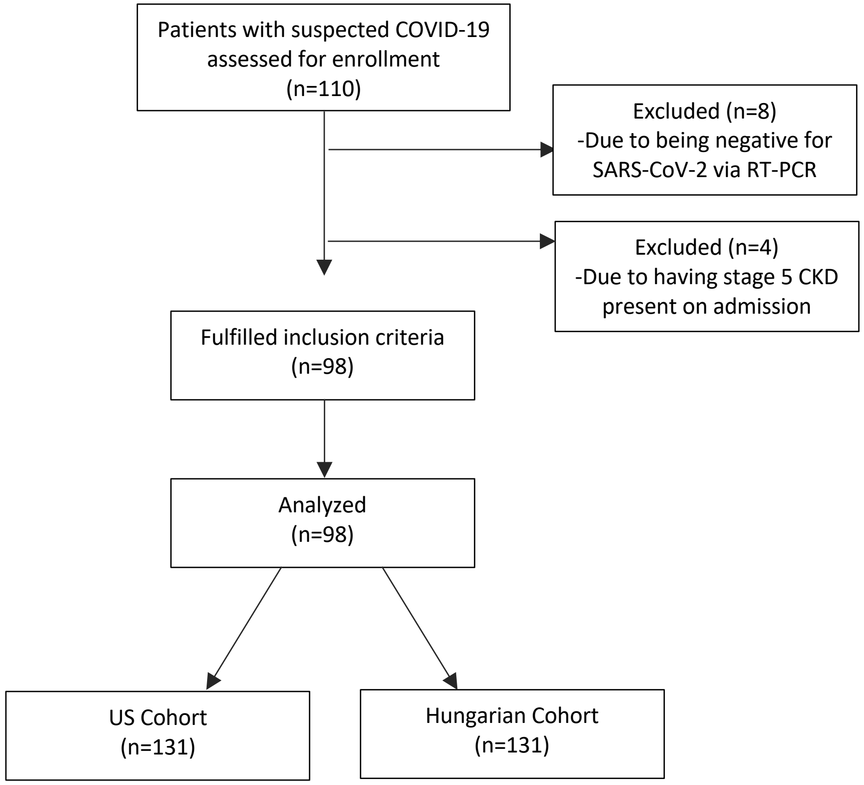

Table S2. Complement levels on admission in patients with COVID-19 in US and Hungary, stratified by development of severe AKI.

Lab variable	Reference range	US Cohort (n=33)			Reference range	Hungarian Cohort (n=98)		
		No severe AKI (n=21)	Severe AKI (n=12)	P-value		No severe AKI (n=87)	Severe AKI (n=11)	P-value

**Alternative pathway**	>63%	89.5 (75.6 – 101.8)	98.9 (80.6 – 104.4)	0.58	70-130%	93 (75.5-102)	68 (58.5-83.5)	**0.008**
**Classical pathway**	101 – 300 CH50/mL	202.0 (140.0 – 252.6)	180.7 (137.1 – 302.8)	0.86	48-103 CH50/mL	74 (66-88.5)	53 (43-64)	**0.001**
**C3**	71 – 150 mg/dL	140.0 (119.0 – 162.0)	139.5 (104.0 – 153.5)	0.58	90-180 mg/dL	129 (109-147)	107 (83-111)	**0.002**
**C3a**	30 - 250 ng/mL	242.0 (169.5 – 392.0)	347.9 (211.9 – 511.6)	0.098	70-270 ng/mL	238 (140-350)	454 (341.5-591)	**0.005**
**C4**	15.7 – 47 mg/dL	29.5 (23.3 – 40.0)	37.1 (30.0 – 45.4)	0.15	15-55 mg/dL	37 (26-49)	26 (17-27)	**0.002**
**C1q**	5.1-7.5 mg/dL	4.3 (3.2 – 6.5)	5.7 (2.4 – 7.8)	0.30	6.0-18.0 mg/dL	10.6 (8.6-13.5)	11.6 (9.6-15.0)	0.31
**sC5b-9**	<244 ng/mL	214.9 (136.2 – 318.5)	201.4 (167.4 – 258.6)	0.75	110-252 ng/mL	287 (203-425)	362 (252-466)	0.28
**Factor B**	13.3-31.5 mg/dL	14.7 (9.4 – 18.1)	17.2 (11.1 – 19.6)	0.22	70-130%	120 (97.5-148)	118 (81-128)	0.16
**Factor H**	37.0 – 68.0 mg/dL	58.7 (48.4 – 78.4)	71.8 (49.4 – 87.0)	0.36	25.0-88.0 mg/dL	74.0 (49.9-102.7)	36.8 (31.9-89.3)	0.12
**Factor I**	2.4 – 4.9 mg/dL	4.7 (3.9 – 5.5)	3.9 (3.2 – 4.8)	0.094	70-130%	103 (84-119)	86 (68-117)	0.27
**C3a/C3**	-	1.65 (1.15-2.38)	2.87 (2.42-3.30)	**0.016**	-	1.79 (1.19-2.76)	3.85 (3.27-6.68)	**<0.001**

All data presented as median (IQR). P-values calculated with Mann-Whitney U test. KDIGO - Kidney Disease Improving Global Outcomes criteria used to define AKI using serum creatinine. No severe AKI – KDIGO 0+1; Severe AKI – KDIGO 2+3; CH50 – 50% hemolytic complement activity. Statistical significance denoted with bold text.
